# Psoriasis and Diabetes: A Multicenter Study in 222078 Type 2 Diabetes Patients Reveals High Levels of Depression

**DOI:** 10.1155/2015/792968

**Published:** 2015-08-17

**Authors:** Anke Schwandt, Dominik Bergis, Albrecht Dapp, Stefan Ebner, Peter M. Jehle, Stefan Köppen, Alexander Risse, Stefan Zimny, Reinhard W. Holl

**Affiliations:** ^1^Institute of Epidemiology and Medical Biometry, Central Institute for Biomedical Technology, German Center for Diabetes Research (DZD), University of Ulm, Albert-Einstein-Allee 41, 89081 Ulm, Germany; ^2^Division of Endocrinology & Metabolism, Department of Internal Medicine I, University Hospital Frankfurt, Theodor-Stern-Kai 7, 60590 Frankfurt, Germany; ^3^Medical Clinic, Health Center Spaichingen, Diabetes Center, Hospital District Tuttlingen, Robert Koch Straße 31, 78549 Spaichingen, Germany; ^4^2nd Department of Internal Medicine, General Hospital Linz, Krankenhausstraße 9, 4021 Linz, Austria; ^5^Department of Internal Medicine, Academic Hospital Paul Gerhardt Stift, Martin Luther University of Halle-Wittenberg, Paul-Gerhardt-Straße 42-45, 06886 Lutherstadt Wittenberg, Germany; ^6^2nd Department of Internal Medicine, Clinical Center HELIOS Hildesheim, Senator-Braun-Allee 33, 31135 Hildesheim, Germany; ^7^Department of Diabetes, Clinical Center Dortmund GmbH, Beurhausstraße 40, 44137 Dortmund, Germany; ^8^Department of General Internal Medicine, Endocrinology and Diabetes, HELIOS Clinic Schwerin, Wismarsche Straße 393-397, 19049 Schwerin, Germany

## Abstract

*Objective*. This study aimed to investigate the association between psoriasis and disease outcome in type 2 diabetes (T2D).* Methods*. 222078 T2D patients (≥10 years old) from the prospective, multicenter diabetes patient registry were analyzed. Specific search items were used to identify psoriasis patients. Multiple regression models were fitted and adjusted for demographic confounder.* Results*. 232 T2D patients had comorbid psoriasis. After adjusting psoriasis patients revealed a higher BMI (31.8 [31.0; 32.6] versus 30.6 [30.5; 30.6] kg/m^2^, *p* = 0.004) and HbA1c (64.8 [62.1; 67.6] versus 59.0 [58.9; 59.1] mmol/mol, *p* < 0.0001). Insulin was used more frequently (62.3 [55.7; 68.5] versus 50.9 [50.7; 51.1] %, *p* = 0.001), only OAD/GLP-1 was similar, and nonpharmacological treatment was less common (13.3 [9.5; 18.3] versus 21.9 [21.7; 22.1] %, *p* = 0.002). Severe hypoglycemia (0.31 [0.238; 0.399] versus 0.06 [0.057; 0.060] events per patient-year, *p* < 0.0001), hypertension (86.1 [81.1; 90.0] versus 68.0 [67.8; 68.2] %, *p* < 0.0001), and thyroid disease (14.0 [10.1; 19.2] versus 4.6 [4.5; 4.7] %, *p* < 0.0001) were more prevalent. Depression occurred more often (10.5 [7.1; 15.2] versus 2.8 [2.7; 2.8] %, *p* < 0.0001). *Conclusions*. Clinical diabetes characteristics in psoriasis T2D patients were clearly worse compared to patients without psoriasis. Comorbid conditions and depression were more prevalent, and more intensive diabetes therapy was required.

## 1. Introduction

Diabetes mellitus is a severe and growing public health problem worldwide [1]. The number of subjects diagnosed with type 2 diabetes (T2D) is increasing [1]. The cause of T2D is multifactorial (genes and epigenetics, insulin resistance, overweight, and physical inactivity).

Previous studies indicated an association between T2D and psoriasis [2–5]. Psoriasis is a multifactorial, chronic immune-mediated inflammatory disorder of the skin, with a genetic component. 2% of the general population are affected [6]. The primary manifestation of the disease is on the skin, although inflammatory processes can occur also in other organs [7]. The plaques are red and infiltrated, covered with a coarse silvery scaling [8]. Often psoriatic lesions affect restricted areas, especially scalp, knees, elbows, or lower back [6]. However, in severe psoriasis, large areas of the body are affected. Therefore, the extent of body involvement, the degree of lesion activity, and the frequency of relapses determine the severity of the disease [6]. Often quality of life is restricted in patients with psoriasis [7, 9]. Psoriasis might influence mental state. Moreover, previous research indicates an association between depression and psoriasis [9, 10].

Chronic inflammation contributes to both T2D and psoriasis [2–4, 11]. The association between the two diseases suggests a pathophysiologic link [3, 4]. In both diseases TH-1 and TH-17 cells are increased [11]. These inflammatory mediators affect diverse processes, such as insulin resistance but also overeating, psychological stress and comorbidities, and release of inflammatory cytokines known to trigger psoriasis [2, 3, 11]. However, Koch et al. [12] reported that no genetic markers of psoriasis were associated with cardiometabolic risk factors and related outcomes, including T2D. A meta-analysis [5] showed that the prevalence and incidence of T2D are increased among psoriasis patients. Moreover, systemic treatments for psoriasis might negatively affect cardiometabolic comorbidities [13].

Psoriasis and its psychosocial symptoms might influence diabetes therapy. Therefore, this study aimed to compare a large number of T2D patients with and without comorbid psoriasis.

Research questions to be answered by this study are as follows:

1. Are there demographic differences between T2D patients with and without psoriasis?
2. Are there differences in diabetes therapy and diabetes-related comorbidities between T2D patients with and without psoriasis? Is there an association between psoriasis and metabolic control?
3. Is the frequency of depression higher in T2D patients with psoriasis?
4. Does psoriasis affect the rate of hypoglycemia, inpatient care, and duration of hospital stay?

## 2. Materials and Methods

### 2.1. Diabetes Patient Registry and Subjects

The analysis is based on the “Diabetes-Patienten-Verlaufsdokumentation” (DPV) developed at the Institute of Epidemiology and Medical Biometry, Ulm, Germany. The Ethics Committee of the University of Ulm has approved the DPV Initiative.

The DPV represents a prospective and multicenter diabetes patient registry [14]. Each center locally documents diabetes-related data. Currently, more than 400 centers from Germany and Austria longitudinally record clinical data of patients with any type of diabetes. This documentation program is standardized and computer based. Every 6 months, the participating centers anonymize the data and transfer them to the University of Ulm, Germany. Inconsistent or implausible data are verified and then aggregated and analyzed.

Until March 2014, 338981 patients with diabetes were documented. As T2D does not occur before puberty, T2D patients over 10 years of age were included. In the current study, 222078 T2D patients aged ≥10 years were analyzed. Clinical data from 323 German and 19 Austrian centers were included. For the present analysis, the most recent treatment year of each patient was aggregated. To identify patients with comorbid psoriasis, specific search items (ICD-10 codes, free text) were used.

### 2.2. BMI and Standard Deviation Score

The body mass index (BMI) corresponds to the body weight in kilograms divided by square of the height in meters (kg/m^2^). As the BMI varies also in adulthood, the age and gender adjusted BMI-SDS by Hemmelmann et al. was calculated based on individual BMI and age- and gender-dependent reference values [14, 15]. Reference values of the Second German National Nutrition Survey were used [15].

### 2.3. Metabolic Control, Diabetes Therapy, and Diabetes-Related Comorbidities

Metabolic control was assessed by hemoglobin A1c (HbA1c). By using the multiple of the mean method (MOM), HbA1c values were mathematically standardized to the reference range of the Diabetes Control and Complications Trial (DCCT, 4.05–6.05% or 20.7–42.6 mmol/mol) [16].

Diabetes therapy was categorized as insulin therapy (insulin therapy alone or in combination with other antidiabetic medications), other antidiabetic medication alone (oral antidiabetic drugs (OAD) or glucagon-like peptide-1 agonist (GLP-1)), or nonpharmacological therapy (lifestyle only). Insulin treatment was categorized as basal supported oral therapy (BOT, only basal insulin), supplementary insulin therapy (SIT, only prandial insulin), conventional insulin therapy (CT, 1–3 injection time points/d), and intensified conventional insulin therapy (ICT, 4–8 injection time points/d or insulin pump therapy).

Insulin dose was calculated per kilogram body weight. The frequency of self-monitoring of blood glucose (SMBG) was recorded per week. Pathological insulin injection sites (e.g., lipohypertrophy) were documented qualitatively (normal/abnormal).

Severe hypoglycemia was defined as an event requiring assistance of another person [17]. The rate of previous inpatient care was determined based on inpatient admissions during the last treatment year. The duration of hospital stay was calculated in days per hospitalization.

Elevated median systolic and/or diastolic blood pressure above 140/90 mmHg or the use of antihypertensive medication was defined as hypertension. Autoimmune thyroid disease was defined by positive thyroid antibodies (MAK/TAK > 100 U/mL) or a clinical diagnosis. Dyslipidemia was classified as the use of lipid-lowering medication and/or at least one lipid parameter on average in the abnormal range (total cholesterol > 5.2 mmol/L, HDL < 0.9 mmol/L, LDL > 3.4 mmol/L, and/or triglycerides > 1.7 mmol/L).

Depression was determined by searching the database for corresponding search items (ICD-10 codes, depression and/or antidepressants). Treatment with steroids was recorded if documented at least once. Steroid treatment was categorized as topical or systemic. Patients are defined as smoker if they report smoking at least one cigarette per day.

### 2.4. Statistical Analysis

All statistical analyses were implemented with Statistical Analysis Software 9.4 (SAS, SAS Institute Inc., Cary, NC, USA).

In observational studies a power analysis is unusual. The large number of patients in the DPV registry ensures that even small differences are statistically significant.

To examine group differences, the following tests were used. Continuous variables were analyzed by Wilcoxon test and binary variables were compared by *χ* ^2^-test. To adjust for multiple testing, *p* values were corrected by Bonferroni-stepdown method (Holm method). Two-sided *p* values ≤0.05 were defined as statistically significant.

To account for potential confounders, regression models were fitted. The models were adjusted for age, sex, and duration of diabetes. Age was categorized into four groups: 10–<40 years, 40–<60 years, 60–<80 years, and ≥80 years. Duration of diabetes was divided into two classes, <2 years and ≥2 years. Modeling the relation between a dependent variable and several explanatory variables, linear regression models were adapted for continuous variables. Residual maximum likelihood estimation technique was used. Equally, logistic regression models were fitted for binary variables. Event rates were analyzed by Poisson regression models with time under risk as offset. Both logistic and Poisson regression were estimated by maximum likelihood estimation. The adjusted estimates (means or proportion with 95% confidence interval) were calculated for each confounding categorical variable based on observed marginal frequencies.

## 3. Results

### 3.1. Description of Study Population

Among the study population of 222078 T2D patients, 232 patients had comorbid psoriasis. 14.7% of psoriasis patients had psoriasis arthritis. The proportion of men was similar in T2D patients with and without psoriasis. The baseline characteristics, classified by presence or absence of psoriasis, are described in Table 1.

**Figure 1:**
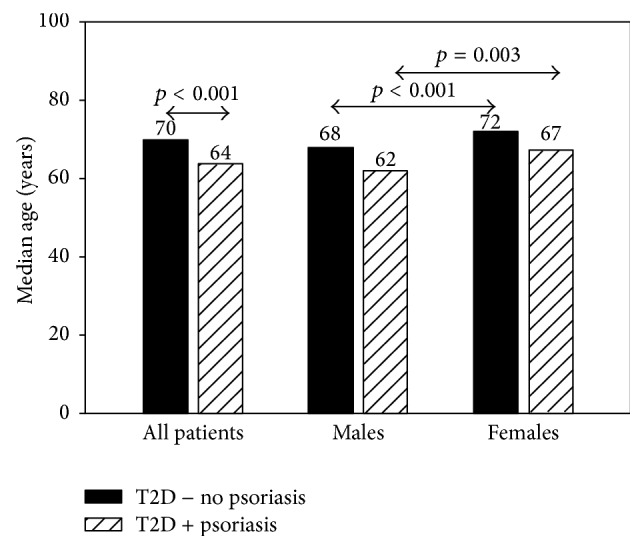
Median age for type 2 diabetes patients with (T2D + psoriasis) and without psoriasis (T2D − no psoriasis) and stratified by gender.

T2D patients with psoriasis were significantly younger compared to patients without psoriasis (Figure 1). The majority of patients in both groups were ≥50 years (psoriasis versus no psoriasis: 87.5 versus 90.7%). Female patients with and without psoriasis were older compared to male patients (Figure 1).

The age of diabetes diagnosis in T2D patients with comorbid psoriasis was significantly younger compared to nonpsoriasis patients (*p* = 0.0001). Diabetes duration was similar between both groups.

Both BMI and BMI-SDS were significantly higher in T2D patients with comorbid psoriasis, even after adjustment for age, sex, and diabetes duration (Table 2). In gender specific analyses, differences in BMI were found for males and females. Statistically significant differences in BMI were found for males only (31.5 [30.5; 32.5] versus 30.2 [30.1; 30.2] kg/m^2^, *p* = 0.009) and not for females (32.1 [30.7; 33.5] versus 30.9 [30.8; 31.0] kg/m^2^, n.s.). With increasing age the BMI decreased. However, significant differences between T2D patients with and without psoriasis were found only in the 60–<80-year-olds (32.2 [31.1; 33.2] versus 30.4 [30.4; 30.5] kg/m^2^).

### 3.2. Metabolic Control and Diabetes Therapy

In Table 2 confounder-adjusted results for BMI, metabolic control, and diabetes therapy in T2D patients with and without psoriasis are summarized. Age, sex, and duration of diabetes were considered as potential confounders.

HbA1c values of T2D patients with comorbid psoriasis were significantly higher compared to T2D patients without psoriasis (*p* < 0.0001). Further adjustment for depression or glucose self-measurements did not alter this finding (depression: 64.8 [62.0; 67.5] versus 59.0 [58.9; 59.1] mmol/mol, *p* < 0.001, and glucose self-measurement: 64.2 [61.1; 67.3] versus 59.2 [59.1; 59.3] mmol/mol, *p* = 0.002). The frequency of self-monitoring of blood glucose per week was significantly higher in psoriasis patients, even after additional adjustment for insulin use (14.8 [13.4; 16.2] versus 12.9 [12.9; 13.0] SMGB per week, *p* = 0.008).

**Figure 2:**
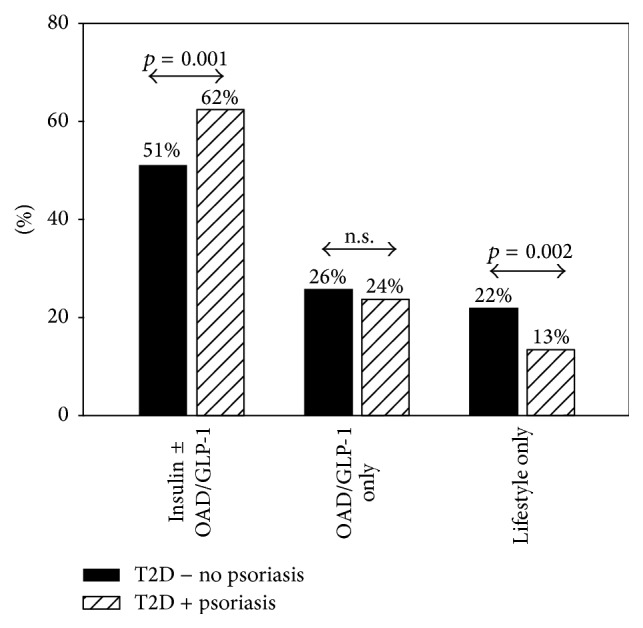
Confounder-adjusted estimates for types of diabetes therapy between type 2 diabetes patients with (T2D + psoriasis) and without comorbid psoriasis (T2D − no psoriasis). Diabetes therapy was insulin therapy, oral antidiabetic medication alone (OAD or GLP-1), or nonpharmacological therapy (lifestyle only).

Figure 2 describes diabetes therapy for T2D patients with and without psoriasis. Insulin therapy was significantly more frequent in patients with psoriasis (*p* = 0.001). In contrast, nonpharmacological therapy was less frequent in T2D patients with psoriasis. Insulin treatment regime was similar in both groups. No differences were observed for insulin dose in insulin-treated patients (0.68 [0.47; 0.90] versus 0.60 [0.59; 0.60] IU/kg). There were no differences between groups in the use of OAD or GLP-1 agonist alone, and metformin use was similar as well.

### 3.3. Diabetes-Related Comorbidities and Complications

Table 3 depicts a confounder-adjusted summary for diabetes-related complications, comorbidities, and comedication, stratified psoriasis.

T2D patients with comorbid psoriasis were more often depressed. Additional adjustment for use of steroids did not alter this finding (9.0 [6.0; 13.2] versus 2.7 [2.6; 2.7]%, *p* < 0.0001). Even after further adjustment for BMI and insulin therapy, the difference remained stable (10.8 [7.2; 15.7] versus 2.7 [2.6; 2.7]%, *p* < 0.0001). Severe hypoglycemia was significantly more common in T2D with psoriasis. Additional adjustment for insulin did not change the significantly higher event rate of severe hypoglycemia in psoriasis patients (0.23 [0.177; 0.297] versus 0.05 [0.048; 0.051] per patient year, *p* < 0.0001). Hypertension was more frequent in patients with psoriasis (*p* < 0.0001) and the use of antihypertensive drugs was more common. A significant difference in autoimmune thyroid disease was observed, with a higher prevalence in T2D patients with comorbid psoriasis (*p* < 0.0001). Psoriasis T2D patients used steroids significantly more often. 50% of T2D patients with comorbid psoriasis were treated with topical steroids and 50% with systemic steroids. The frequency of smokers was higher in T2D patients with psoriasis (13.3 [9.3; 18.6] versus 9.3 [9.1; 9.5]%, *p* = 0.05). In T2D patients with psoriasis, previous inpatient care was more frequent and the duration of hospital stay was longer.

Although a high frequency of dyslipidemia was observed, it was comparable between groups. No difference could be observed for pathological insulin injection sites (10.2 [6.1; 16.6] versus 10.8 [10.5; 11.0]%).

## 4. Discussion

This prospective, multicenter study based on a large T2D population (*n* = 222078) aimed to investigate the association between psoriasis and disease outcome in T2D. Comorbid psoriasis is associated with worse metabolic control, more diabetes-related complications, and a higher rate of depression. Differences in diabetes therapy were observed between both groups. Therefore, patients with comorbid psoriasis might be a disadvantaged subgroup of T2D patients.

Insulin therapy was used significantly more often in T2D patients with comorbid psoriasis, while nonpharmacological therapy was less common. These results strengthen a previous study indicating that patients with T2D and psoriasis have been treated more often pharmacologically compared to T2D patients without psoriasis [3]. Azfar et al. [3] reported that psoriasis T2D patients are more likely to receive insulin as well as oral antihyperglycemic medication. Moreover, psoriasis is considered a T-cell-mediated inflammatory disorder, which is characterized by both activation of antigen-presenting cells and expansion of TH-1 and TH-17 lymphocytes [8, 11]. This high level of inflammation induces insulin resistance, leading to reduced signaling of the insulin receptors [2, 3, 8, 11, 18]. The interaction between inflammation and insulin resistance may explain the fact that insulin is required more often for the treatment of T2D patients with psoriasis requiring insulin more often.

Metformin is the first choice of oral antidiabetic medication in T2D [19, 20]. In this study, one-third of both T2D patients with and without psoriasis were treated with metformin.

Metabolic control was worse in T2D patients with comorbid psoriasis. This may be probably due to reduced insulin sensitivity in psoriasis patients [2, 3, 11, 18]. A further reason might be the higher BMI in this patient group. Moreover, psoriasis T2D patients have to manage two diseases and thus the burden is increased. Therefore, diabetes self-care might be neglected despite requirement of more intensive medical care for T2D patients with psoriasis. This is in line with our finding of a higher hospitalization rate in this patient group. In contrast to previous studies [21, 22] reporting a relationship between metabolic control and depression in T2D patients, in this research the difference of HbA1c could not be explained by depression only.

In our study, the use of steroids was higher in T2D patients with psoriasis. Steroid therapy is one key therapeutic option for psoriasis with good efficacy [23]. In line with German psoriasis guidelines, in this study 50% of psoriasis T2D patients with documented steroid use were treated locally [24], while the remaining used systemic steroids. The high rate of systemic steroids might be explained in part by psoriasis patients with an arthritic component. However, previous studies reported that treatment with steroids is associated with obesity, dyslipidemia, insulin resistance, glucose intolerance, and also diabetes [25]. Therefore, steroid use might contribute to worse metabolic control in psoriasis patients and also to a high frequency of metabolic syndrome.

Depression occurred three times more frequently in T2D patients with comorbid psoriasis compared to patients without psoriasis. It is well known that psoriasis patients experience psychosocial difficulties such as depression, anxiety, and avoidance of social activities due to living with a chronic, disfiguring condition and fear from rejections by other persons, especially due to their visible psoriatic lesions [10, 26, 27]. Griffiths and Richards [10] reported that an individual's emotional state might influence the development of psoriasis. Furthermore, a meta-analysis concluded that there is a bidirectional relationship between depression and T2D [28]. Mezuk et al. [28] reported that there is a strong association between depression and the incidence of T2D. However, in T2D patients there was a modest increased risk of depression only. Moreover, Khalid et al. [2] reported that antidepressant medication was increased in diabetes patients with psoriasis compared to the reference population without psoriasis. Moreover, previous studies reported that steroid use was often linked to mood changes [29]. Contrary to Warrington and Bostwick [30] who reported an association between depression and steroid use, in the present research, the difference of depression between patients with and without comorbid psoriasis could not be explained by steroid use.

The rate of severe hypoglycemia was significantly higher in psoriasis patients. Contrary to other studies, the difference could not be explained by insulin therapy [31]. However, a previous study reported that depression is a positive predictor of hypoglycemia in T2D patients [32]. One reason might be hypoglycemia unawareness due to depression [33]. This corresponds to our finding of a higher frequency of depression in T2D patients with comorbid psoriasis which may in turn contribute to hypoglycemia.

In this research, the frequency of autoimmune thyroid disease was more prevalent in T2D patients with psoriasis. Both psoriasis and thyroid disease are related to autoimmunity [34]. However, this fact has not been well established and is discussed controversially. Antonelli et al. [35] reported a higher prevalence of thyroid autoimmunity in psoriasis arthritis patients. In contrast, Gul et al. [36] described no difference for thyroid disease between psoriasis patients without the arthritis component and healthy individuals. Reasons for the different results might be diverse definitions of psoriasis and different patient populations as well as ethnical background. Furthermore, previous studies reported a relationship between thyroid disease and T2D [37–39]. In both diseases endocrinopathies might influence each other in multiple ways [37]. Worse controlled diabetes might affect thyroid metabolism, and vice versa thyroid disease might deteriorate glycemia [37].

Several studies reported a relationship between metabolic syndrome and both T2D and psoriasis [40, 41]. This is in line with our finding of a high rate of hypertension, obesity, and dyslipidemia. In many studies hypertension was associated with psoriasis [26, 42, 43] but also with T2D [44, 45]. Investigations from Armesto et al. [4] confirmed our finding of a higher prevalence of hypertension in psoriasis T2D patients. Moreover, previous research indicated an association between psoriasis and a greater likelihood of hypertension, especially in severely affected psoriasis patients [43]. Moreover, BMI and BMI-SDS were significantly higher in psoriasis patients. Previous studies indicated an association between obesity and both psoriasis [46] and T2D [44, 45]. In the analysis of female sex, the lack of significance for BMI might be due to the small number of patients in this subgroup. In line with our finding, in both T2D and psoriasis research a higher risk of dyslipidemia was observed [47, 48].

The frequency of smoking was higher in T2D patients with comorbid psoriasis. It is well known that smoking is associated with an increased risk of T2D [49]. Moreover, a previous study reported that the prevalence of smoking was higher in psoriasis patients compared to control patients [50]. Setty et al. [51] indicated an increased risk for developing psoriasis in past and current smokers. Furthermore, higher intensity and longer duration of smoking were associated with increased clinical severity of psoriasis [52].

The major strengths of the present study are the large number of T2D patients and the large number of diabetes care institutions included in the database. Furthermore, a wide age range was studied. One limitation is that psoriasis diagnosis may be underreported in the diabetes registry. In addition, there were no data assessing psoriasis severity. Moreover, only data of diabetes care institutions could be included, not from dermatological care facilities. Therefore, incomplete documentation of psoriasis in the database could not be totally ruled out. Moreover, definitions of depression and steroid use are based on documentation provided by health care professionals. However, potential underreporting could not be totally eliminated. In addition, treatment with steroids could not be studied in more detail. A further limitation might be that in psoriasis patients additional steroid use may affect metabolic control. No further adjustment for physical activity was possible due to the small number of psoriasis T2D patients with documented physical activity (*n* = 7). Moreover, detailed information on dietary intake is not available in the database as the assessment of dietary intake in real-life is difficult.

## 5. Conclusion

Clear differences between T2D patients with and without comorbid psoriasis could be demonstrated by this study. Disease outcome in T2D patients with comorbid psoriasis was clearly worse. Moreover, differences in diabetes therapy were observed. Psoriasis patients required a more intensive diabetes therapy. Therefore, physicians should focus on comorbid diseases such as psoriasis in T2D patients. Since an earlier intervention may improve the psoriasis outcome, a more intensive screening is important. Particularly in Germany, every second psoriasis patient is medically undersupplied and is not treated according to current guidelines [53]. T2D patients with comorbid psoriasis should be further encouraged to improve their metabolic status in order to lower the risk for complications. Lifestyle changes such as physical activity showed a positive influence on metabolic control, diabetes treatment, BMI, and cardiovascular risk profile in T2D patients [54]. Moreover, previous research indicated that physical activity is an effective treatment in psoriasis [55].

## Figures and Tables

**Table 1:** Baseline characteristics of the study population, classified by presence or absence of psoriasis.

	Type 2 diabetes			*p* value
	All	No psoriasis	Psoriasis	
*N*	222078	221846	232	—
Age, years	69.7 [60.1; 77.3]	69.7 [60.1; 77.3]	63.7 [55.7; 71.3]	<0.0001
Male sex, %	51.4	51.4	58.2	n.s.
Age at diabetes diagnosis, years	58.4 [48.6; 68.1]	58.4 [48.6; 68.1]	54.5 [47.1; 61.9]	0.0001
Diabetes duration, years	8.1 [2.7; 14.7]	8.1 [2.7; 14.7]	7.3 [2.3; 13.7]	n.s.

Data are given as median with quartiles. Wilcoxon and *χ* ^2^ test were used for the comparison between patients with and without psoriasis. n.s.: not significant.

**Table 2:** Confounder-adjusted estimates for BMI, metabolic control, and diabetes therapy in type 2 diabetes patients with or without psoriasis.

Adjusted estimates	Type 2 diabetes		*p* value
	No psoriasis	Psoriasis	
*N*	221846	232	—

BMI, kg/m^2^	30.6 [30.5; 30.6]	31.8 [31.0; 32.6]	0.004
	(*n* = 196674)	(*n* = 205)	
BMI-SDS	0.44 [0.44; 0.45]	0.68 [0.52; 0.84]	0.005
	(*n* = 198925)	(*n* = 203)	

Metabolic control			
HbA1c, mmol/mol	59.0 [58.9; 59.1]	64.8 [62.1; 67.6]	<0.0001
	(*n* = 200030)	(*n* = 219)	

Type of diabetes therapy			
Insulin, %	50.9 [50.7; 51.1]	62.3 [55.7; 68.5]	0.001
OAD/GLP-1 only, %	25.7 [25.5; 25.9]	23.7 [18.7; 29.6]	n.s.
Nonpharmacological therapy, %	21.9 [21.7; 22.1]	13.3 [9.5; 18.3]	0.002
Subgroup OAD			
Metformin, %	32.9 [32.7; 33.1]	36.7 [30.8; 43.0]	n.s.
Insulin treatment regime			
CT, %	29.6 [29.3; 29.9]	24.5 [17.8; 32.8]	n.s.
	(*n* = 113780)	(*n* = 114)	
ICT or pump, %	43.9 [43.6; 44.2]	44.5 [36.5; 52.8]	n.s.
SIT, %	11.7 [11.5; 11.9]	11.3 [7.1; 17.5]	n.s.
BOT, %	12.1 [11.9; 12.3]	16.4 [11.2; 23.5]	n.s.

Glucose self-monitoring			
SMBG, per week	12.9 [12.8; 12.9]	16.0 [14.4; 17.7]	0.0001
	(*n* = 171865)	(*n* = 176)	

Adjusted means or proportion with 95% confidence intervals. The number in parentheses indicates the patients studied. Data were adjusted for age, sex, and duration of diabetes. n.s.: not significant, BMI: body mass index, BMI-SDS: body mass index standard deviation score, HbA1c: hemoglobin A1c, OAD/GLP-1: oral antidiabetic drug/glucagon-like peptide-1 agonist, CT: conventional insulin therapy, ICT: intensified conventional insulin therapy, SIT: supplementary insulin therapy, BOT: basal supported oral therapy, and SMBG: self-monitoring of blood glucose.

**Table 3:** Confounder-adjusted summary for diabetes-related complications, comorbidities, and comedication, stratified by psoriasis.

Adjusted estimates	Type 2 diabetes		*p* value
	No psoriasis	Psoriasis	
*N*	221846	232	—
Depression, %	2.8 [2.7; 2.8]	10.5 [7.1; 15.2]	<0.0001
Hypertension, %	68.0 [67.8; 68.2]	86.1 [81.1; 90.0]	<0.0001
	(*n* = 208211)	(*n* = 226)	
Autoimmune thyroid disease, %	4.6 [4.5; 4.7]	14.0 [10.1; 19.2]	<0.0001
	(*n* = 221815)	(*n* = 232)	
Dyslipidemia, %	82.5 [82.3; 82.7]	80.8 [74.2; 85.9]	n.s.
	(*n* = 168168)	(*n* = 186)	
Severe hypoglycemia, per patient year	0.06 [0.057; 0.060]	0.31 [0.238; 0.399]	<0.0001
	(*n* = 221836)	(*n* = 232)	
Previous inpatient care, %	10.5 [10.4; 10.6]	20.2 [15.5; 26.0]	<0.0001
Duration of hospital stays, days per hospitalization	4.19 [4.18; 4.20]	7.67 [7.26; 8.11]	<0.0001

Comedication			
Antihypertensive drugs, %	50.2 [50.0; 50.4]	76.5 [70.7; 81.4]	<0.0001
Steroids, %	1.5 [1.4; 1.5]	6.3 [3.8; 10.3]	<0.0001

Adjusted means or proportions with 95% confidence interval. Potential confounders are age, sex, and duration of diabetes. The number in parentheses indicates the patients studied. n.s.: not significant.

## References

[1] International Diabetes Federation (IDF) (2014). *Diabetes Atlas*.

[2] Khalid U., Hansen P. R., Gislason G. H. (2013). Psoriasis and new-onset diabetes: a Danish nationwide cohort study. *Diabetes Care*.

[3] Azfar R. S., Seminara N. M., Shin D. B., Troxel A. B., Margolis D. J., Gelfand J. M. (2012). Increased risk of diabetes mellitus and likelihood of receiving diabetes mellitus treatment in patients with psoriasis. *Archives of Dermatology*.

[4] Armesto S., Santos-Juanes J., Galache-Osuna C., Martinez-Camblor P., Coto E., Coto-Segura P. (2012). Psoriasis and type 2 diabetes risk among psoriatic patients in a Spanish population. *Australasian Journal of Dermatology*.

[5] Armstrong A. W., Harskamp C. T., Armstrong E. J. (2013). Psoriasis and the risk of diabetes mellitus: a systematic review and meta-analysis. *JAMA Dermatology*.

[6] Christophers E. (2001). Psoriasis—epidemiology and clinical spectrum. *Clinical and Experimental Dermatology*.

[7] Augustin M., Spehr C., Radtke M. A. (2014). German psoriasis registry PsoBest: objectives, methodology and baseline data. *Journal der Deutschen Dermatologischen Gesellschaft*.

[8] Boehncke W.-H., Boehncke S. (2014). More than skin-deep: the many dimensions of the psoriatic disease. *Swiss Medical Weekly*.

[9] Palijan T. Ž., Kovačević D., Koić E., Ružić K., Dervinja F. (2011). The impact of psoriasis on the quality of life and psychological characteristics of persons suffering from psoriasis. *Collegium Antropologicum*.

[10] Griffiths C. E. M., Richards H. L. (2001). Psychological influences in psoriasis. *Clinical and Experimental Dermatology*.

[11] Davidovici B. B., Sattar N., Jörg P. C. (2010). Psoriasis and systemic inflammatory diseases: potential mechanistic links between skin disease and co-morbid conditions. *Journal of Investigative Dermatology*.

[12] Koch M., Baurecht H., Ried J. S. (2015). Psoriasis and cardiometabolic traits: modest association but distinct genetic architectures. *Journal of Investigative Dermatology*.

[13] Gisondi P., Galvan A., Idolazzi L., Girolomoni G. (2015). Management of moderate to severe psoriasis in patients with metabolic comorbidities. *Frontiers in Medicine*.

[14] Scheuing N., Bayer C., Best F. (2013). Is there a benefit to use calculated percent body fat or age- and gender-adjusted BMI-SDS(LMS) to predict risk factors for cardiovascular disease? A German/Austrian multicenter DPV-Wiss analysis on 42 048 type 2 diabetic patients. *Experimental and Clinical Endocrinology & Diabetes*.

[15] Hemmelmann C., Brose S., Vens M., Hebebrand J., Ziegler A. (2010). Percentiles of body mass index of 18–80-year-old German adults based on data from the second national nutrition survey. *Deutsche Medizinische Wochenschrift*.

[16] American Diabetes Association (2007). Consensus statement on the worldwide standardisation of the HbA1c measurement. *Diabetologia*.

[17] Seaquist E. R., Anderson J., Childs B. (2013). Hypoglycemia and diabetes: a report of a workgroup of the American Diabetes Association and the Endocrine Society. *Diabetes Care*.

[18] Gyldenløve M., Storgaard H., Holst J. J., Vilsbøll T., Knop F. K., Skov L. (2015). Patients with psoriasis are insulin resistant. *Journal of the American Academy of Dermatology*.

[19] Pfeiffer A. F., Klein H. H. (2014). The treatment of type 2 diabetes. *Deutsches Ärzteblatt International*.

[20] Deutsche Diabetes Gesellschaft Evidenzbasierte Nationale Versorgungsleitlinien. http://www.deutsche-diabetes-gesellschaft.de/leitlinien/evidenzbasierte-leitlinien.html.

[21] Papelbaum M., Moreira R. O., Coutinho W. (2011). Depression, glycemic control and type 2 diabetes. *Diabetology and Metabolic Syndrome*.

[22] Richardson L. K., Egede L. E., Mueller M., Echols C. L., Gebregziabher M. (2008). Longitudinal effects of depression on glycemic control in veterans with Type 2 diabetes. *General Hospital Psychiatry*.

[23] Weigle N., Mcbane S. (2013). Psoriasis. *The American Family Physician*.

[24] Nast A., Boehncke W. H., Mrowietz U. (2012). S3—guidelines on the treatment of psoriasis vulgaris. *Journal der Deutschen Dermatologischen Gesellschaft*.

[25] van Raalte D. H., Ouwens D. M., Diamant M. (2009). Novel insights into glucocorticoid-mediated diabetogenic effects: towards expansion of therapeutic options?. *European Journal of Clinical Investigation*.

[26] Baeta I. G. R., Gontijo B., Bittencourt F. V., Goulart E. M. A. (2014). Comorbidities and cardiovascular risk factors in patients with psoriasis. *Anais Brasileiros de Dermatologia*.

[27] Schäfer I., Rustenbach S. J., Radtke M., Augustin J., Glaeske G., Augustin M. (2011). Epidemiology of psoriasis in Germany—analysis of secondary health insurance data. *Gesundheitswesen*.

[28] Mezuk B., Eaton W. W., Albrecht S., Golden S. H. (2008). Depression and type 2 diabetes over the lifespan: a meta-analysis. *Diabetes Care*.

[29] Brown E. S. (2009). Effects of glucocorticoids on mood, memory, and the hippocampus: treatment and preventive therapy. *Annals of the New York Academy of Sciences*.

[30] Warrington T. P., Bostwick J. M. (2006). Psychiatric adverse effects of corticosteroids. *Mayo Clinic Proceedings*.

[31] Zammitt N. N., Frier B. M. (2005). Hypoglycemia in type 2 diabetes: pathophysiology, frequency, and effects of different treatment modalities. *Diabetes Care*.

[32] Tschöpe D., Bramlage P., Binz C., Krekler M., Deeg E., Gitt A. K. (2012). Incidence and predictors of hypoglycaemia in type 2 diabetes—an analysis of the prospective DiaRegis registry. *BMC Endocrine Disorders*.

[33] Murata G. H., Duckworth W. C., Shah J. H., Wendel C. S., Hoffman R. M. (2004). Factors affecting hypoglycemia awareness in insulin-treated type 2 diabetes: the Diabetes Outcomes in Veterans Study (DOVES). *Diabetes Research and Clinical Practice*.

[34] Nograles K. E., Brasington R. D., Bowcock A. M. (2009). New insights into the pathogenesis and genetics of psoriatic arthritis. *Nature Clinical Practice Rheumatology*.

[35] Antonelli A., Delle Sedie A., Fallahi P. (2006). High prevalence of thyroid autoimmunity and hypothyroidism in patients with psoriatic arthritis. *Journal of Rheumatology*.

[36] Gul U., Gonul M., Kaya I., Aslan E. (2009). Autoimmune thyroid disorders in patients with psoriasis. *European Journal of Dermatology*.

[37] Witting V., Bergis D., Sadet D., Badenhoop K. (2014). Thyroid disease in insulin-treated patients with type 2 diabetes: a retrospective study. *Thyroid Research*.

[38] Díez J. J., Sánchez P., Iglesias P. (2011). Prevalence of thyroid dysfunction in patients with type 2 diabetes. *Experimental and Clinical Endocrinology and Diabetes*.

[39] Radaideh A.-R. M., Nusier M. K., Amari F. L. (2004). Thyroid dysfunction in patients with type 2 diabetes mellitus in Jordan. *Saudi Medical Journal*.

[40] LeRoith D., Cohen D. H. (2000). Metabolic syndrome. *Endotext South Dartmouth (MA)*.

[41] Alsufyani M. A., Golant A. K., Lebwohl M. (2010). Psoriasis and the metabolic syndrome. *Dermatologic Therapy*.

[42] Takahashi H., Iizuka H. (2012). Psoriasis and metabolic syndrome. *Journal of Dermatology*.

[43] Takeshita J., Wang S., Shin D. B. (2015). Effect of psoriasis severity on hypertension control: a population-based study in the United Kingdom. *JAMA Dermatology*.

[44] Stumvoll M., Goldstein B. J., van Haeften T. W. (2005). Type 2 diabetes: principles of pathogenesis and therapy. *The Lancet*.

[45] Cukierman-Yaffe T., Gerstein H. C., Williamson J. D., et al (2009). Relationship between baseline glycemic control and cognitive function in individuals with type 2 diabetes and other cardiovascular risk factors: the action to control cardiovascular risk in diabetes-memory in diabetes (ACCORD-MIND) trial. *Diabetes Care*.

[46] Engin B., Kutlubay Z., Yardimci G. (2014). Evaluation of body composition parameters in patients with psoriasis. *International Journal of Dermatology*.

[47] Prey S., Paul C., Bronsard V. (2010). Cardiovascular risk factors in patients with plaque psoriasis: a systematic review of epidemiological studies. *Journal of the European Academy of Dermatology and Venereology*.

[48] Chehade J. M., Gladysz M., Mooradian A. D. (2013). Dyslipidemia in type 2 diabetes: prevalence, pathophysiology, and management. *Drugs*.

[49] Willi C., Bodenmann P., Ghali W. A., Faris P. D., Cornuz J. (2007). Active smoking and the risk of type 2 diabetes: a systematic review and meta-analysis. *The Journal of the American Medical Association*.

[50] Herron M. D., Hinckley M., Hoffman M. S. (2005). Impact of obesity and smoking on psoriasis presentation and management. *Archives of Dermatology*.

[51] Setty A. R., Curhan G., Choi H. K. (2007). Smoking and the risk of psoriasis in women: Nurses' Health Study II. *American Journal of Medicine*.

[52] Fortes C., Mastroeni S., Leffondré K. (2005). Relationship between smoking and the clinical severity of psoriasis. *Archives of Dermatology*.

[53] Berufsverband der Deutschen Dermatologen (2014). *Unterversorgung kennt keine Grenzen*.

[54] Hermann G., Herbst A., Schütt M. (2014). Association of physical activity with glycaemic control and cardiovascular risk profile in 65 666 people with type 2 diabetes from Germany and Austria. *Diabetic Medicine*.

[55] Wilson P. B., Bohjanen K. A., Ingraham S. J., Leon A. S. (2012). Psoriasis and physical activity: a review. *Journal of the European Academy of Dermatology and Venereology*.

